# Imaging for the detection and evaluation of pulmonary edema: state of the art

**DOI:** 10.1590/0100-3984.2025.58.e5-en

**Published:** 2025-10-06

**Authors:** Thiago Krieger Bento da Silva, Matheus Zanon

**Affiliations:** 1 Department of Radiology, Hospital São Lucas, Pontifícia Universidade Católica do Rio Grande do Sul (PUCRS), Porto Alegre, RS, Brazil; 2 Pavilhão Pereira Filho, Santa Casa de Porto Alegre, Porto Alegre, RS, Brazil

Pulmonary edema, a common clinical condition, particularly in intensive care and emergency settings, is the result of multiple pathophysiological mechanisms and etiologies. Rapid, accurate identification of edema is crucial for patient management, a process in which imaging plays a central role. Chest X-ray, computed tomography (CT), and lung ultrasound are fundamental diagnostic tools, allowing not only the detection of edema but also the inference of its likely cause based on characteristic morphological patterns^(1)^.

Radiologists routinely encounter infiltrative pulmonary opacities, among which edema must always be considered. This diagnostic challenge is compounded by an aging population with increasing comorbidities and complex therapies, further underscoring the critical importance of imaging.

The article authored by Dias et al. and entitled “Pulmonary edema: what can it tell us? A pictorial essay”^(2)^, recently published in Radiologia Brasileira, offers a concise, well-structured review of the main mechanisms of pulmonary edema formation and correlates them with the most typical findings on chest X-ray, CT, and ultrasound. The article is useful for the training and continuing education of radiologists, pulmonologists, intensivists, and clinicians. One of the main merits of the essay is precisely the clear organization of the content into thematic sections, complemented by representative, high-quality images to highlight the different presentation patterns of pulmonary edema, facilitating the understanding of the radiological patterns. Highlights include the discussion of forms of edema with less obvious causes, such as neurogenic, post-transplant, post-nonfatal-drowning, and re-expansion edema.

Future directions for the literature in this field include multimodal integration, such as using a combination of imaging methods (chest X-ray, CT, ultrasound, and magnetic resonance imaging) with quantitative analysis and imaging biomarkers, for automated analysis of the signs of pulmonary congestion and for risk stratification^(3)^. The literature also indicates a growing role for lung ultrasound in critical settings, driven by technological advances, its satisfactory accuracy, and its utility for bedside assessment^(4)^. This trend may reduce reliance on plain radiographs for diagnosis in such environments. That makes it even more important for radiologists to be aware of this trend, particularly as we increasingly observe CT being utilized for patients who respond poorly to initial treatment based on ultrasound findings. This may be a point of difficulty in decision-making, because we previously had serial X-rays showing a sequence of events, which facilitated image-based decision-making.

Finally, future studies could explore multimodal integration among CT, ultrasound, and emerging techniques such as the use of artificial intelligence, aiming not only at the detection but also at the automated etiological classification of pulmonary edema^(5)^. This is a rapidly evolving field that will continue to transform clinical and diagnostic practice. It should be borne in mind that technology alone is not enough: critical thinking will continue to be required; and radiologists will increasingly need to master the tools and, above all, lead the discussion, indicating when and how to use each method, avoiding both overand under-investigation.
